# Oral and gut dysbiosis leads to functional alterations in Parkinson’s disease

**DOI:** 10.1038/s41531-022-00351-6

**Published:** 2022-07-07

**Authors:** Sungyang Jo, Woorim Kang, Yun Su Hwang, Seung Hyun Lee, Kye Won Park, Mi Sun Kim, Hyunna Lee, Hyung Jeong Yoon, Yoo Kyoung Park, Mauricio Chalita, Je Hee Lee, Hojun Sung, Jae-Yun Lee, Jin-Woo Bae, Sun Ju Chung

**Affiliations:** 1 Department of Neurology, Asan Medical Center, University of Ulsan College of Medicine, Seoul, 05505 South Korea; 2 Department of Biology and Department of Life and Nanopharmaceutical Sciences, Kyung Hee University, Seoul, 02447 South Korea; 3 CJ Bioscience Inc, Seoul, 04527 South Korea; 4 Department of Neurology, Uijeongbu Eulji Medical Center, Eulji University School of Medicine, Uijeongbu-si, Gyeonggi-do 11759 South Korea; 5 Bigdata Research Center, Asan Institute for Life Sciences, Asan Medical Center, Seoul, 05505 South Korea; 6 Department of Medical Nutrition, Graduate School of East-West Medical Science, Kyung Hee University, Yongin, Gyeonggi-do 17104 South Korea

**Keywords:** Parkinson's disease, Parkinson's disease

## Abstract

Although several studies have identified a distinct gut microbial composition in Parkinson’s disease (PD), few studies have investigated the oral microbiome or functional alteration of the microbiome in PD. We aimed to investigate the connection between the oral and gut microbiome and the functional changes in the PD-specific gut microbiome using shotgun metagenomic sequencing. The taxonomic composition of the oral and gut microbiome was significantly different between PD patients and healthy controls (*P* = 0.003 and 0.001, respectively). Oral *Lactobacillus* was more abundant in PD patients and was associated with opportunistic pathogens in the gut (FDR-adjusted *P* < 0.038). Functional analysis revealed that microbial gene markers for glutamate and arginine biosynthesis were downregulated, while antimicrobial resistance gene markers were upregulated in PD patients than healthy controls (all *P* < 0.001). We identified a connection between the oral and gut microbiota in PD, which might lead to functional alteration of the microbiome in PD.

## Background

Parkinson’s disease (PD) is a chronic progressive neurodegenerative disease affecting more than 6 million people worldwide^1^. The non-motor symptoms of PD, such as constipation, impaired olfaction, and rapid eye movement sleep behavior disorders, are frequently present before the onset of motor symptoms, which might be explained by the accumulation of alpha-synuclein in the peripheral nervous system before spreading to the substantia nigra^2^. The progressive spreading of alpha-synuclein from peripheral nerves into the brain was previously proposed in neuropathologic studies and also demonstrated in mouse models, where injection of preformed alpha-synuclein fibrils into the gastric muscular layers resulted in the spread of pathologic alpha-synuclein into the brain, while truncal vagotomy prevented the spread^3,4^. These findings led to the hypothesis that the pathology of PD may initiate in the gut^5^.

Animal studies have clarified the role of the gut microbiome in PD. Germ-free mice showed limited alpha-synuclein pathology, while mice with microbiota from PD patients exhibited enhanced motor dysfunction^6^. Human studies have also revealed distinct gut microbial compositions associated with PD^7–11^, but little is known about the alteration of the oral microbiome in PD. Dysbiosis of the oral microbiome has been observed in systemic diseases including cancer, autoimmune diseases, and gastrointestinal diseases^12^. The systemic effects of oral dysbiosis can be explained by local and systemic inflammation as well as the oral-gut connection. In general, the oral bacteria poorly colonize in a healthy digestive system^13,14^. However, in pathological circumstances, oral pathogens could affect the gut environment, enhancing the oral-gut dysbiosis connection. For example, an increased abundance of the oral-associated microbiome was observed in the gut in patients with inflammatory bowel disease, colon cancer, and liver cirrhosis^12,15,16^. Oral dysbiosis could also affect the pathogenesis of PD through the oral-gut connection. However, the number of studies on the oral microbiome in PD is limited, especially on its link with the gut microbiome^5^.

In addition, prior investigations have focused on the analysis of bacterial composition in PD using 16 S rRNA gene-based amplicon sequencing^17^. This method only targets the 16 S rRNA locus, which is a taxonomically informative marker^18^. Although this sequencing method is inexpensive and analytically convenient^19^, it has low taxonomic resolution at the genus level, and only enables indirect assumptions about biological functions^20–22^. Functional analysis of the microbiome is essential to determine how the bacterial composition affects the development of PD and to use it as a therapeutic target. In addition, the ecological dynamics of the microbiota enable the recovery of initial function, despite compositional changes^23^. Whole-genome shotgun metagenomic sequencing evaluates DNA from the whole microbial community^18^, thus providing high-resolution detection at the species level and elucidation of biological functions encoded by the microbial genome^18,21,22^. The use of whole-genome shotgun sequencing is increasing, but few studies have been conducted in PD patients to date^24^.

In the present study, we aimed to address the lack of data on the link between the oral and gut microbiome in PD, and on the functional alterations of the PD microbiota. We investigated the taxonomic and functional changes in the oral and gut microbiota in PD patients and healthy controls. In addition, we identified a discriminatory panel of candidate microbial biomarkers using the oral and gut microbiota.

## Results

### Baseline clinical characteristics of PD patients and healthy controls

The study population included 91 patients with PD and 85 healthy controls (HCs). The mean age at study enrollment was not significantly different between the PD patients and HCs (mean ± standard deviation, 65.1 ± 7.9 vs. 64.6 ± 8.0, *P* = 0.66) (Table 1). The prevalence of constipation was higher in PD patients than in HCs (47.3% vs. 12.9%, *P* < 0.001). Sex, body mass index, and dietary intake were not significantly different between two groups. The degrees of swallowing difficulty and olfactory dysfunction were higher in PD patients than in HCs. In PD patients, the median disease duration (IQR) was 2.0 (0.0─6.0) years and the median (IQR) Unified PD Rating Scale (UPDRS) part 3 was 32.0 (25.0─40.0).

**Table 1:** Baseline clinical characteristics of patients with Parkinson’s disease (PD) and healthy controls

	PD patients (*n* = 91)	HCs (*n* = 85)	*P* value
Age, years	65.1 ± 7.9	64.6 ± 8.0	0.66
Male	49 (53.8%)	40 (47.1%)	0.45
BMI, kg/m^2^	24.3 (22.3 − 26.4)	23.9 (21.3 − 25.6)	0.09
Education, years	12.0 (12.0 − 16.0)	12.0 (12.0 − 16.0)	0.88
Clinical symptoms			
IBS	3 (3.3%)	(3.5%)	>0.99
Constipation	43 (47.3%)	11 (12.9%)	<0.001*
Bristol stool scale	4.0 (2.0 − 4.0)	4.0 (4.0 − 4.0)	<0.001*
Swallowing test	0.0 (0.0 − 3.0)	0.0 (0.0 − 0.0)	0.003*
Smell test	9.0 (6.0 − 10.0)	10.0 (10.0 − 10.0)	<0.001*
Disease duration	2.0 (0.0 − 6.0)	—	
UPDRS			
Part 1	5.0 (3.0 − 7.0)	—	
Part 2	7.0 (4.0 − 11.0)	—	
Part 3	32.0 (25.0 − 40.0)	—	
Part 4	0.0 (0.0 − 2.0)	—	
Total score	44.0 (34.0 − 60.0)	—	
Hoehn and Yahr stage	2.0 (2.0 − 3.0)	—	
Medication for PD			
Levodopa	74 (81.3%)	—	
Dopamine agonist	35 (38.5%)	—	
COMT inhibitor	7 (7.7%)	—	
MAO-B inhibitor	25 (27.5%)	—	
Amantadine	19 (20.9%)	—	
Levodopa equivalent daily dose, mg	450.0 (275.0 − 802.5)	—	
Daily dietary intake	(*n* = 83)	(*n* = 78)	
Total energy, kCal	2168.5 ± 500.5	2160.3 ± 566.6	0.92
Carbohydrate, g	295.1 ± 69.9	288.4 ± 79.6	0.57
Protein, g	88.2 ± 25.8	88.9 ± 25.1	0.85
Animal source	47.2 ± 15.5	47.1 ± 16.5	0.96
Plant source	40.9 ± 16.5	41.8 ± 16.4	0.73
Fat, g	68.5 ± 26.4	67.7 ± 24.2	0.84
Animal source	40.2 ± 20.0	38.7 ± 18.4	0.63
Plant source	28.3 ± 12.9	29.0 ± 12.8	0.75
Fiber, g	38.4 ± 17.0	38.0 ± 17.6	0.87
Soluble	5.0 ± 2.5	5.1 ± 2.4	0.08
Insoluble	18.9 ± 8.1	19.2 ± 8.6	0.80

Values are mean ± standard deviation, *n* (%), or median (interquartile range).

*PD* Parkinson’s disease, *HCs* healthy controls, *BMI* body mass index, *IBS* irritable bowel syndrome, *UPDRS* Unified Parkinson’s Disease Rating Scale, *COMT* catechol-O-methyltransferase, *MAO-B* monoamine oxidase B.

*P < 0.05.

Previous studies indicated that PD medication such as catechol-o-methyl transferase (COMT) inhibitors and anticholinergics affected the microbiota composition, so we conducted a beta-diversity analysis to detect any effect of PD medication on the microbiota composition. Stool microbiome was related to the use of COMT inhibitor (*P* = 0.001) and amantadine (*P* = 0.048), while the oral microbiome was related to the use of dopamine agonist (*P* = 0.003) and amantadine (*P* = 0.049).

### 16 S rRNA gene sequencing of the oral and gut microbiota

Stool samples were analyzed in 88 PD patients and 84 HCs, and oral samples were analyzed in 74 PD patients and 69 HCs after excluding those with low sequence coverage and those who refused to give oral samples (Supplementary Fig. 1). Characteristics of the subset of patients sampled for oral and gut microbiome were similar to those of the total study population (Supplementary Table 1 and Supplementary Table 2).

**Fig. 1:**
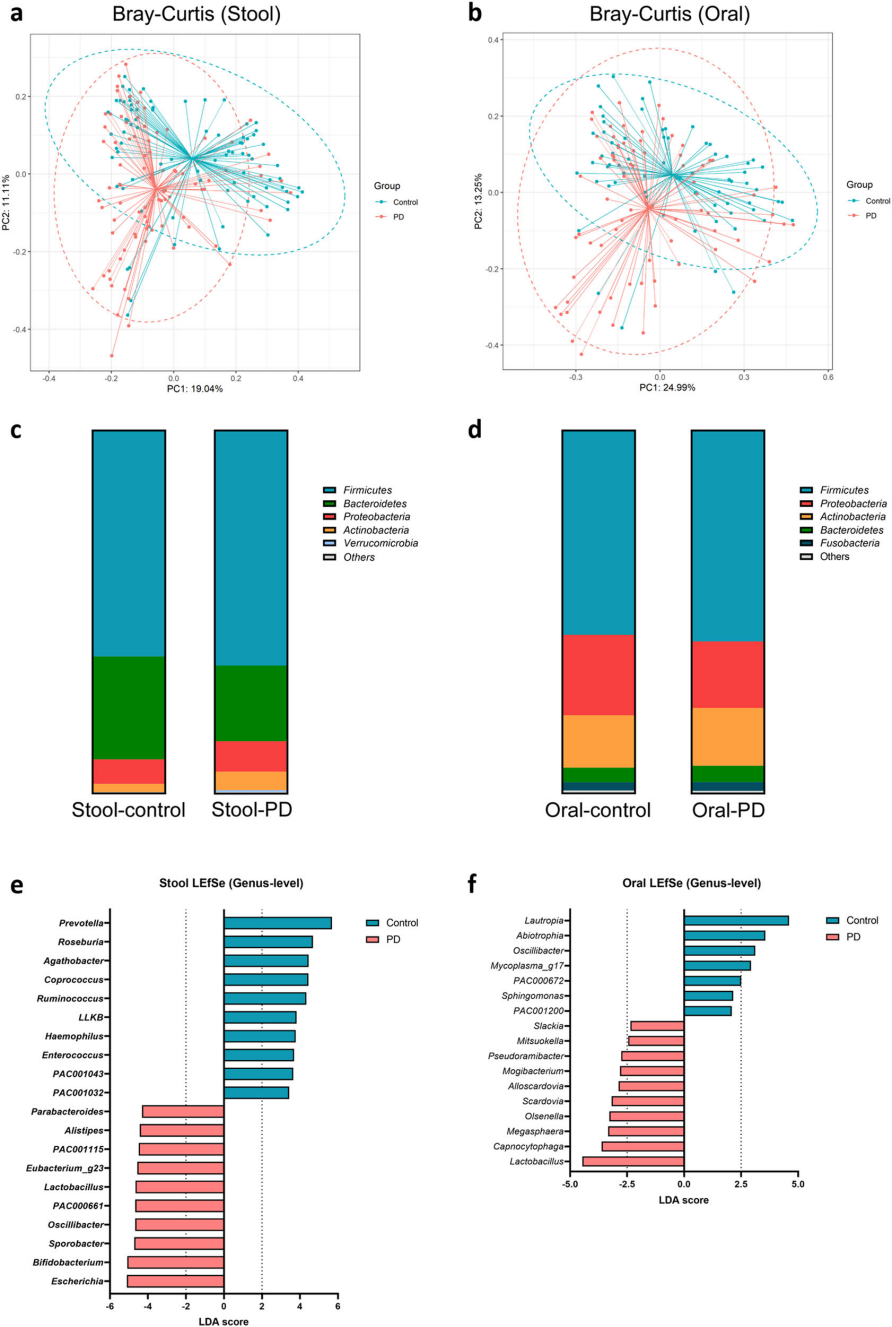
Gut and oral microbial community structure based on 16 S rRNA gene sequencing. **a**, **b** Principal coordinates analysis based on the Bray-Curtis dissimilarity between Parkinson’s disease (PD) and healthy controls in **a** stool and **b** oral samples. **c**, **d** Phylum-level bar charts of PD and HCs in the **c** stool and **d** oral samples. **e**, **f** Comparison of the **e** gut microbiome and **f** oral microbiome between PD and HCs using genus-level LEfSe analysis. The top 10 genera based on the LDA score in the PD and control groups are shown. The cut-off value for oral LDA effect size was set at 2.0. *LLKB* (*Lachnospiraceae*; LLKB01000001), *PAC001043* (*Lachnospiraceae*, AJ576336), *PAC001032* (*Lachnospiraceae*, unpublished), *PAC001115* (*Christensenellaceae*, HQ716403) *PAC000661* (*Oscillospiraceae*; JN713389) *PAC002046* (*Lachnospiraceae*, GQ897562), and *Eubacterium*_g23 (*Oscillospiraceae*, GQ502529) were the phylotype genera (Family, NCBI accession number).

Beta-diversity analysis showed significant differences in the microbial composition between the two groups in the gut (*P* = 0.001) and oral samples (*P* = 0.003) (Fig. 1a, b), while there were no significant differences in alpha diversity (Supplementary Fig. 2).

In the gut, the most common phylum was *Firmicutes*, which had a similar abundance between PD patients and HCs. *Bacteroidetes* was significantly more abundant in HCs (FDR-adjusted *P; Q* = 0.007), while *Proteobacteria* was more abundant in PD patients (*Q* = 0.048) (Fig. 1c). In the oral samples, the most common phylum was *Firmicutes*, followed by *Proteobacteria* and *Actinobacteria*, all of which did not show significant differences between the two groups (Fig. 1d).

According to the linear discriminant analysis (LDA) effect size (LEfSe) analysis, short-chain fatty acid-producing gut bacteria such as *Prevotella*, *Roseburia*, *Coprococcus*, and *Ruminococcus* had lower LDA scores in PD patients. *Escherichia*, *Bifidobacterium*, *Sporobacter*, *Oscillibacter*, and *Lactobacillus* had higher LDA scores in PD patients than in HCs (Fig. 1e). Aside from the top 20 genera according to the LEfSe analysis, genera that were abundant in patients with PD in previous studies (i.e., *Hungatella*, *Odoribacter*, *Alistipes*, and *Collinsella*) were also more abundant in PD patients than in HCs (*Q* < 0.030)^25–27^, along with the proinflammatory genus *Mogibacterium* (*Q* = 0.043). In the oral samples of PD patients, *Lautropia*, *Abiotrophia*, and *Oscillibacter* had lower LDA scores while *Lactobacillus*, *Capnocytophaga*, and *Megasphaera* had higher LDA scores than in HCs (Fig. 1f).

We compared the gut microbiota between patients whose H&Y stage was less than 3 (mild PD) and patients whose H&Y stage was 3 or greater (severe PD). The beta-diversity was not significantly different between mild PD and severe PD (*P* = 0.147) (Supplementary Fig. 3). LEfSe analysis showed that the PD-associated microbiome in this study, such as *Lactobacillus* and *Bifidobacterium*, and gut pathogens such as *Klebsiella*, were higher in severe PD compared with mild PD. We also compared the oral microbiota between mild PD and severe PD. The beta diversity was significantly different between mild PD and severe PD (*P* = 0.042). In the severe PD group, oral *Hemophilus* and *Lactobacillus* were increased, and *Lautropia* was decreased.

### Functional analysis based on 16 S rRNA gene sequencing

We indirectly compared the function of the oral microbiome using 16 S rRNA gene sequencing between patients with PD and HCs using PICRUSt. Beta-glucoside operon transcriptional antiterminator was higher in the oral samples of PD compared with the healthy controls (Supplementary Table 3). However, the results were not statistically significant after adjusting for multiple comparisons.

### Association between the oral and gut microbiome

In the previous four studies that investigated the oral microbiome in PD and in this study, one common oral bacteria consistently found to be increased in PD was *Lactobacillus*^14,28–31^. Therefore, we focused on oral *Lactobacillus*, a facultative anaerobe that could survive in the digestive tract.

**Fig. 2:**
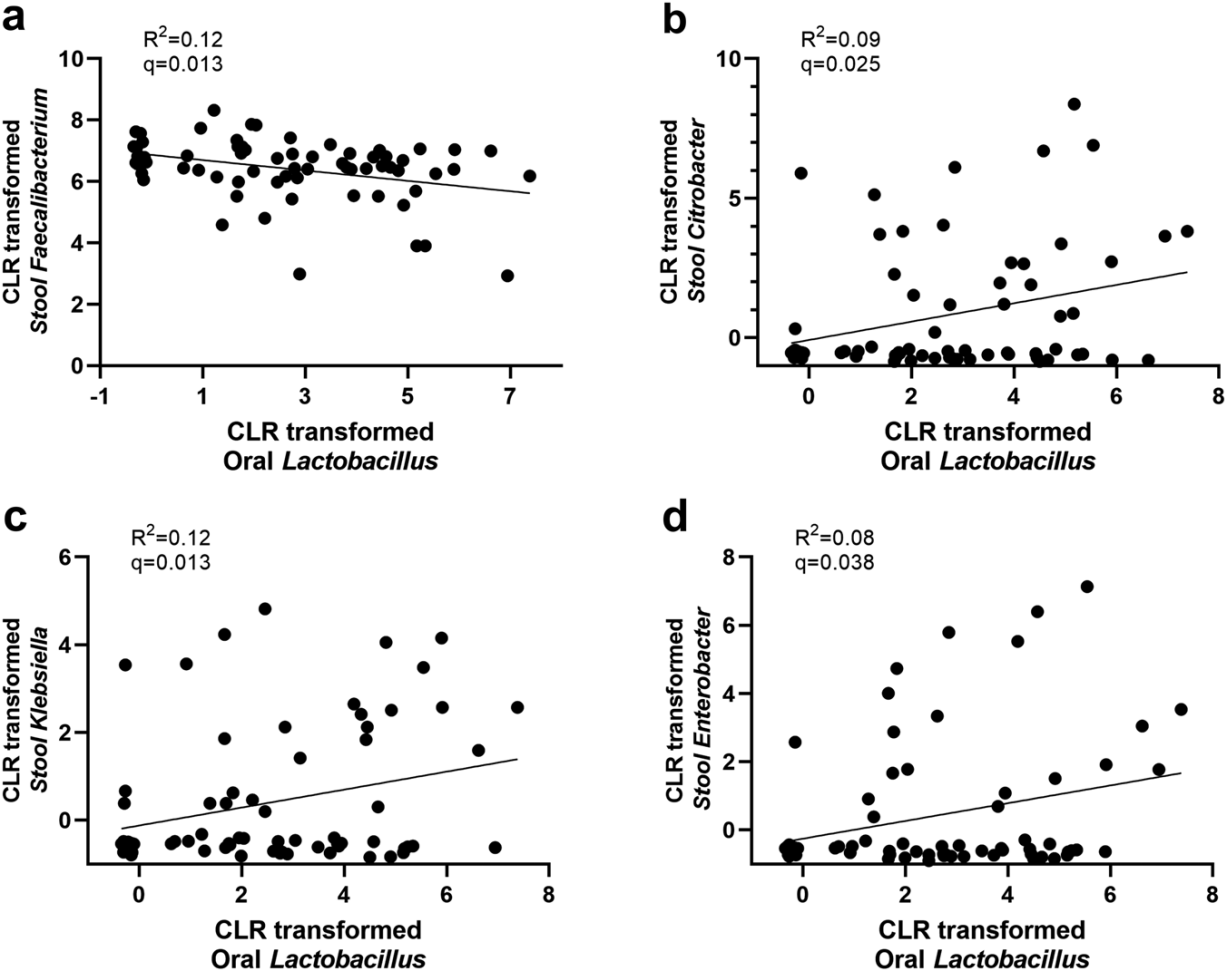
Correlation between oral *Lactobacillus* and stool bacteria in Parkinson’s disease. **a**–**d** Linear regression analysis between oral *Lactobacillus* and stool. **a** *Faecalibacterium*, **b** *Citrobacter*, **c** *Klebsiella*, and **d** *Enterobacter*.

*Lactobacillus* was significantly more abundant in PD patients, in both the oral cavity (*Q* = 0.044) and the gut (*Q* = 0.187 and *P* = 0.049). We examined the correlation between the abundance of oral *Lactobacillus* in the mouth with that of gut microbiome. In PD patients, the abundance of oral *Lactobacillus* was associated with a low abundance of gut *Faecalibacterium*, which is a representative intestinal commensal bacterium (*Q* = 0.013), and with a high abundance of gut *Citrobacter*, *Klebsiella*, and *Enterobacter*, all of which belong to the opportunistic pathogenic *Enterobacteriaceae* (*Q* ≤ 0.038) (Fig. 2). However, the correlation was not significant in HCs. Oral *Lactobacillus* and stool *Lactobacillus* did not show a significant association in both PD and HCs (*Q* = 0.36).

### Association between the microbiome and clinical manifestations of PD

Canonical correspondence analysis showed that PD severity index (UPDRS total score) was positively correlated with the disease duration, dysphagia, and the use of COMT inhibitor or amantadine, whereas age, Bristol stool index, IBS, and olfactory function were not significantly correlated with PD severity (Supplementary Fig. 4 and Supplementary Table 4). The total UPDRS score was associated with a higher abundance of *Bifidobacterium* (*Q* = 0.002) and *Lactobacillus* (*Q* = 0.006) among the top 20 genera from the LEfSe analysis. However, there was no genus correlated negatively with the PD severity indexes. Stool firmness (low Bristol stool scale) was related to a low abundance of *Prevotella* (*Q* = 0.003). The use of COMT inhibitor or amantadine was positively associated with disease severity. In contrast, none of the oral microbiota was significantly correlated with the clinical symptoms of PD.

### Whole-genome shotgun metagenomics of the gut microbiome

**Fig. 3:**
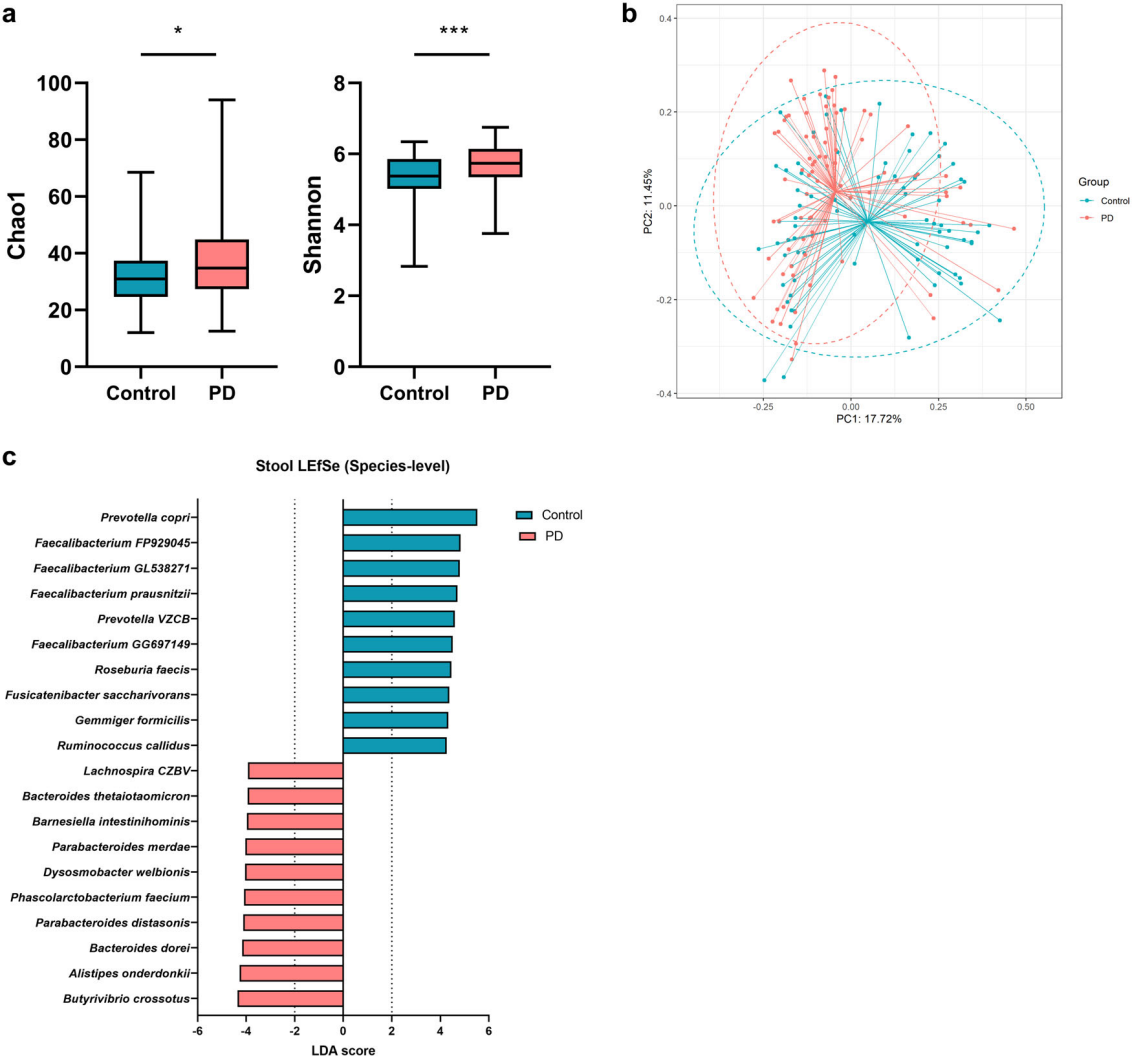
Gut microbial species-level community structures based on whole-genome shotgun sequencing. **a** Alpha diversity between Parkinson’s disease (PD) and healthy controls. **b** Principal coordinates analysis based on the Bray-Curtis dissimilarity. **c** Species-level LEfSe analysis. The top 10 genera in the gut based on the LDA score in the Parkinson’s disease and HC groups are shown. *Faecalibacterium FP929045* (AJ270470), *Faecalibacterium GL538271* (GL538271), *Prevotella VZCB* (VZCB01000106), *Faecalibacterium GG697149* (GG697149) and *Lachnospira CZBV* (CZBV01000019) were phylotype species (NCBI accession number). Boxplot centerline represents the median (50th percentile). The top and bottom hinges represent 75th and 25th percentiles, respectively. The upper and lower whiskers correspond to the highest and lowest data points. n.s: not significant, *P < 0.05, **P < 0.01, ***P < 0.001.

Using stool samples, we conducted high-resolution taxonomic and functional analyses based on the whole-genome shotgun metagenomics data. The alpha diversity was significantly higher in PD patients than in HCs (*P* = 0.048 for Chao1, and *P* = 0.0003 for Shannon) (Fig. 3a), and the beta-diversity was also significantly different between the two groups (*P* = 0.001) (Fig. 3b). We found that *Prevotella copri*, *Prevotella VZCB*, and four *Faecalibacterium* species were less abundant in PD patients than in HCs (Fig. 3c). Conversely, possible pathogens or proinflammatory species, such as *Alistipes onderdonkii*, *Bacteroides dorei*, *Parabacteroides merdae*, and *Butyrivibrio crossotus*, were more abundant in PD patients. Aside from the top 20 species according to the LEfSe analysis, proinflammatory species (*Bacteroides cellulosilyticus*, *Bacteroides eggerthii*, *Butyricimonas virosa*, and *Bilophila wadsworthia*) were also significantly more abundant in PD patients (*Q* < 0.041).

### Functional analysis based on whole-genome shotgun metagenomics

**Fig. 4:**
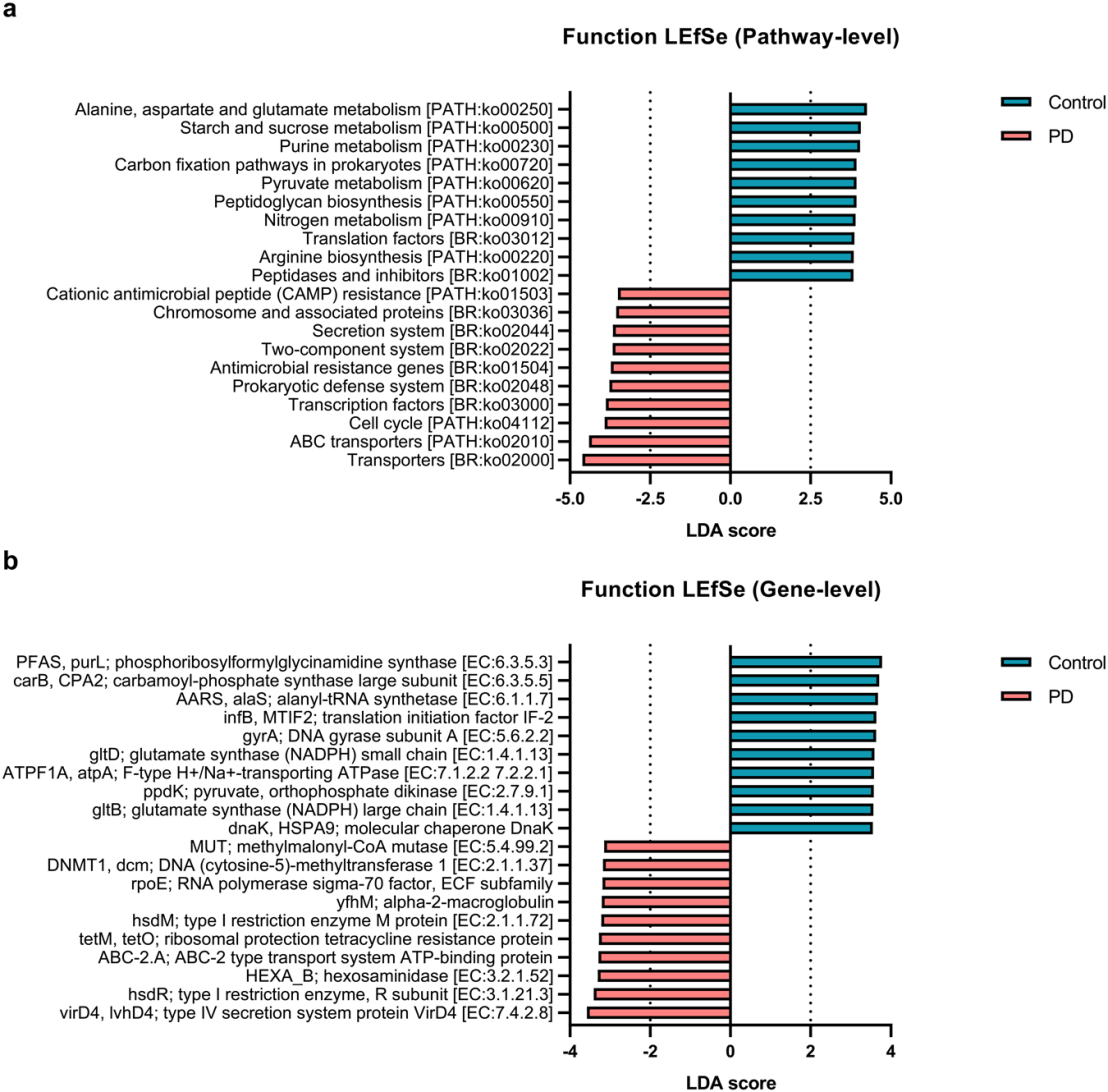
Gut microbial functional profiles based on shotgun sequencing. **a**, **b** Comparison of gut microbial functional profiles between patients with Parkinson’s disease (PD) and healthy controls in **a** pathway-level, and **b** gene-level.

Microbial genes involved in glutamate metabolism (ko00250) and arginine biosynthesis (ko00220) were downregulated in PD patients compared with HCs (all *Q* < 0.0001) (Fig. 4). Conversely, microbial genes involved in the prokaryotic defense system (ko02048), antimicrobial resistance (ko01504), and cationic antimicrobial peptide (CAMP) resistance (ko01503) were upregulated in PD patients compared with HCs. Interestingly, glutamate decarboxylase, which converts L-glutamate into γ -aminobutyric acid (GABA), was present at a higher frequency in the gut of PD patients than in HCs (*Q* = 0.004). Conversely, glutamate synthase and phosphoribosylformylglycinamidine synthase, all of which produce L-glutamate, were present at a lower frequency in PD patients than in HCs (all *Q* < 0.0001) (Supplementary Fig. 5). Furthermore, genes involved in the synthesis of arginine (ornithine carbomoyltransferase, *Q* = 0.021; argininosuccinate synthase, *Q* < 0.0001; and argininosuccinate lyase, *Q* < 0.0001) were present at a lower frequency in PD patients than in HCs, and genes involved in the consumption of arginine (arginase, *Q* < 0.0001) were present at a higher frequency in PD patients than in HCs.

**Fig. 5:**
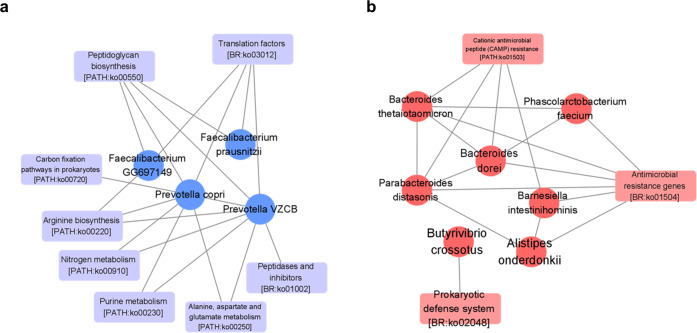
Network analysis showing the correlations between gut bacterial species and function. **a**, **b** Network analysis of the 20 selected species and associated functional pathways. The nodes between functions were eliminated and only the edges that have significant correlations are shown. The minimum R value cut-off was 0.35. The red color represents species or functions that were less prevalent in Parkinson’s disease (PD) patients, and the blue colors represent those that were more prevalent in PD patients.

Network analysis showed that alanine, aspartate, and glutamate metabolism (ko00250) and arginine metabolism (ko00220) were strongly correlated with *Prevotella copri*, *Prevotella VZCB*, and *Faecalibacterium* species (all *Q* < 0.0001) (Fig. 5). Prokaryotic defense system (ko02048), antimicrobial resistance (ko01504), and CAMP resistance (ko01503) genes were significantly correlated with *Phascolarctobacterium faecium* (*Q* < 0.0001), *Bacteroides thetaiotaomicron* (*Q* < 0.0001), *Bacteroides dorei* (*Q* < 0.0001), *Parabacteroides distasonis* (*Q* < 0.0001), *Barnesiella intestinihominis* (*Q* < 0.0001) and *Alistipes onderdonkii* (*Q* < 0.0001).

### Machine learning analysis for the diagnosis of PD

**Fig. 6:**
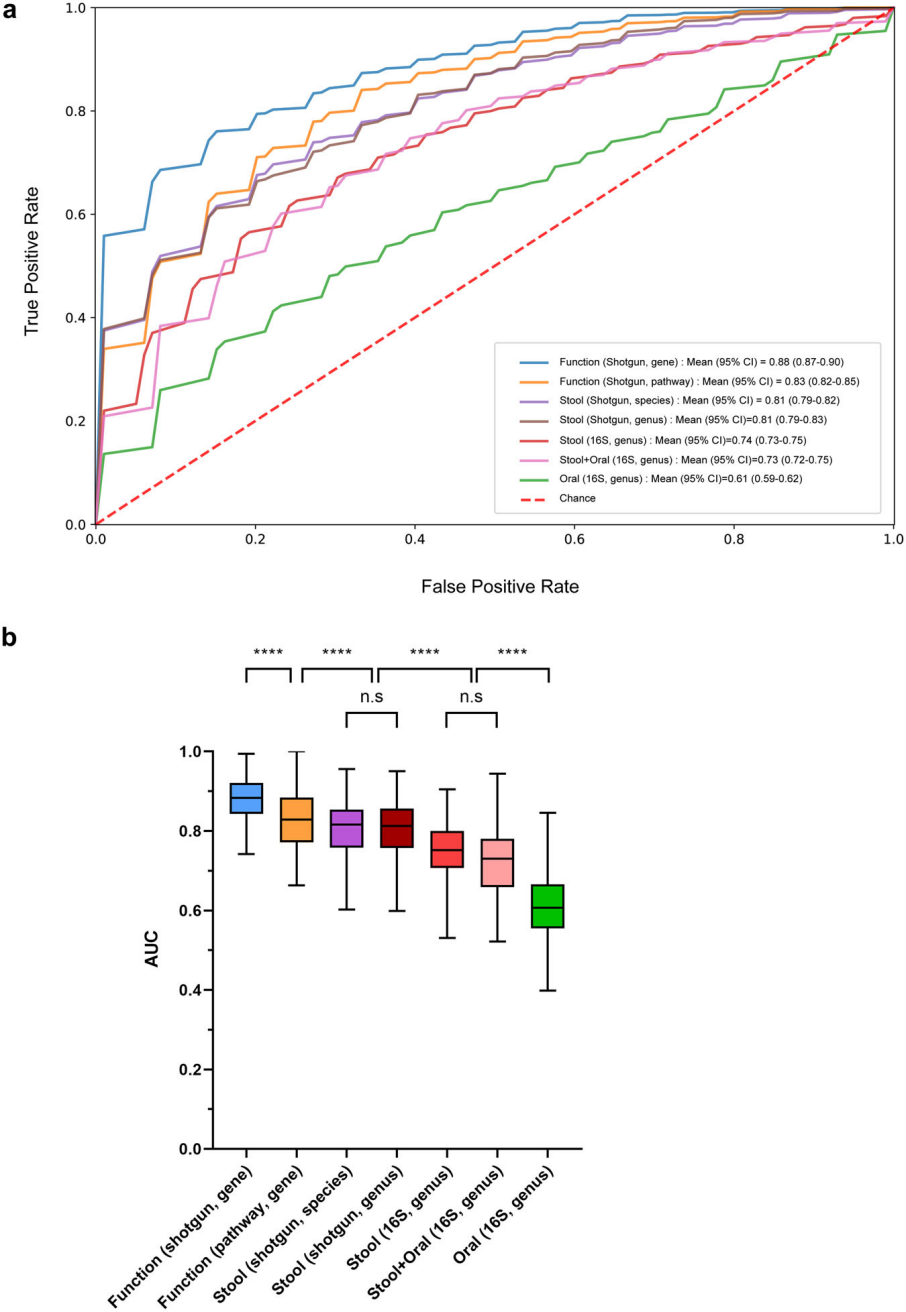
Discriminating Parkinson’s disease from healthy controls based on the oral and gut microbiome. **a** Receiver operating characteristic curve of random forest classifier using shotgun or 16 S rRNA gene sequencing. **b** Comparison of each classifier. Boxplot centerline represents the median (50th percentile). The top and bottom hinges represent 75th and 25th percentiles, respectively. The upper and lower whiskers correspond to the highest and lowest data points. n.s: not significant, *P < 0.05, **P < 0.01, ***P < 0.001, ****P < 0.0001.

We trained random forest classifiers with bacterial composition (oral, genus level; stool, species and genus level) and function (gene and pathway level). The random forest classifier using gene markers from whole-genome shotgun metagenomic sequencing had the highest area under the curve (AUC) for discriminating PD patients from HCs (0.88, 95% CI 0.87–0.90), followed by the classifiers using functional pathways (AUC = 0.83, 95% CI 0.82–0.85) and species-level taxa (AUC = 0.81, 95% CI 0.79–0.82) from shotgun sequencing (Fig. 6). The most commonly used functional features in the classifier were genes involved in arginine and glutamate biosynthesis, which is consistent with the results of the functional analysis (Supplementary Fig. 6). The classifier using genus-level data from shotgun sequencing had an AUC of 0.81, higher than that using genus-level data from 16 S rRNA gene sequencing (AUC = 0.74, *P* < 0.0001). Combining the gut and oral microbiome did not significantly improve the discriminatory performance compared to the gut microbiome alone (AUC = 0.73 vs. 0.74, *P* = 0.63).

## Discussion

In the present study, we investigated the connection between the oral and gut microbiome in PD patients, and found that oral *Lactobacillus*, which was more abundant in PD patients than in HCs, was positively correlated with opportunistic pathogens in the gut. We also identified distinct taxonomic and functional signatures associated with PD. Microbial gene markers for glutamate and arginine biosynthesis were downregulated in PD patients compared with HCs, while antimicrobial resistance genes were upregulated in PD patients. A random forest classifier using the functional gene markers from shotgun metagenomic sequencing was superior to one based on taxonomic markers from 16 S rRNA gene sequencing at distinguishing PD patients from HCs.

The oral microbiome from patients with PD had a distinct taxonomic composition compared with healthy controls. Oral *Lactobacillus* had the highest LDA score in PD patients than in HCs, and it did not show a significant correlation with confounding factors, including disease duration and dysphagia. In the previous four studies that investigated the oral microbiome in PD and in this study, the one common oral bacteria consistently increased in PD was *Lactobacillus*^14,28–30^. The other oral bacteria showed variability among studies; for example, *Capnocytophaga* was decreased in PD in the study of Pereira et al.^28^, but it was increased in PD in this study. However, the mechanism by which oral *Lactobacillus* affects the pathogenesis of PD remains unclear. Commensal bacteria in the oral cavity include *Streptococcus*, *Fusobacterium*, *Haemophilus*, and *Prevotella* species, while oral *Lactobacillus* is abundant in dental caries^32^. Oral pathogens could affect systemic disease through local and systemic inflammation^33^, or they could reach the stomach from the oral cavity and colonize the intestine^34^. Interestingly, it was hypothesized that *Lactobacillus reuteri* might increase the release of alpha-synuclein into the enteric nervous system by increasing the firing rate of mesenteric afferent nerve bundles^35,36^.

Oral bacteria are poor colonizers in a healthy digestive system^13,14^. However, oral-associated microbiota were found in the gut of patients with inflammatory bowel disease, colon cancer, and liver cirrhosis^12,15,16^. We can assume that in pathological circumstances, oral pathogens can colonize the gut, or dead bacteria could affect gut microbiota growth by acting as a nutritional source^13,14^. In this study, we found that the increase in oral *Lactobacillus* was associated with an increase in gut pathogens, but only in PD, not in healthy controls. Oral *Lactobacillus* could migrate to the gut and alter the intestinal environment to promote the colonization of pathogens, as it may do in gastrointestinal disease^12,15,16^. This implies an oral-gut microbiome connection in pathological conditions involving damage to the enteric nervous system and decreased gastrointestinal motility, as is found in PD. The oral microbiota is an attractive diagnostic and therapeutic target because it is easily accessible and modifiable. Additional studies are required to elucidate the association between oral bacteria, especially *Lactobacillus* species, and the pathogenesis of PD.

Oral *Lactobacillus* and stool *Lactobacillus* did not show a significant association in either PD or HCs. This might be explained by the fact that the main *Lactobacillus* species in the oral cavity and the intestine are different, even though *Lactobacillus* is a facultative anaerobe that could survive in the intestine. For example, *Lactobacillus rhamnosus, Lactobacillus casei, Lactobacillus fermentum*, and *Lactobacillus salivarius* are oral *Lactobacillus* species most commonly found in healthy young people^32^, while *Lactobacillus paracaesei, Lactobacillus ruminis*, and *Lactobacillus casei* are gut lactobacillus species most commonly observed in healthy older adults^37^. Because stool *Lactobacillus* was higher in PD, and it showed a significant correlation with disease severity, oral and stool *Lactobacillus* would be expected to show some correlation when using species-level analysis.

The microbiome is an ecosystem in which numerous bacterial taxa exist, and it maintains stability and resilience through functional redundancy^23,38,39^. Although current approaches to studying the PD microbiome are based on determining the bacterial composition, the function of the ecosystem may not change in responses to differences in the bacterial composition. This is reflected in the result of the random forest classifier in the present study, which showed that gene markers and functional pathways had better discriminating performance than taxonomic composition. Therefore, functional approaches are important when investigating the effect of the microbiome on systematic diseases.

We indirectly compared the function of the oral microbiome using 16 S rRNA gene sequencing between patients with PD and healthy controls, but the results were not statistically significant after adjusting for multiple comparisons. Beta-glucoside operon transcriptional anti-terminator showed higher trends in the oral samples of PD. It is produced by many species in the oral microbiome, and *Lactobacillus* has high beta-glucosidase activity^40,41^. Orally secreted beta-glucosidase was associated with halitosis and dental biofilm, possibly mediated through a volatile organic compound.

Whole-genome shotgun metagenomic sequencing enabled precise functional analysis of the gut microbiome in PD patients, which is not possible using traditional 16 S RNA sequencing data. Gut microbial genes in PD patients were related to high consumption and low production of glutamate and arginine, which were also the most frequently used features in the random forest classifier. Decreased glutamate in the serum and stool samples of patients with PD has been observed in previous studies^25,42,43^. Intestinal cells use glutamate to produce energy and protect the mucosa through glutathione, and decreased glutamate may thus contribute to mucosal damage. In addition, glutamate in the gut environment reduces inflammation by inducing the differentiation of naïve T cells into regulatory T cells^44^. Arginine reduces gut inflammation and pathology, and is important for normal brain function^45^. The upregulation of antimicrobial resistance genes observed in PD patients suggests prior infection and the use of antibiotics, and this is consistent with a previous finding that the use of antibiotics increases the risk of PD^46^. CAMP is an essential defense system for the host against infection^47^. The upregulation of CAMP resistance genes in the gut microbiome of PD implies increased antimicrobial peptide secretion and inflammation in the host. The gut inflammation promotes alpha-synuclein aggregation in the enteric nerve, or conversely, the accumulation of alpha-synuclein may provoke inflammation and dysbiosis^48^. Therefore, our data showed functional alteration of the gut microbiome in PD, which could contribute to the pathogenesis of PD.

Shotgun metagenomic sequencing detected PD-associated microbial species that were not found by 16 S rRNA gene sequencing. The PD-associated microbial species included pathogenic or proinflammatory species, such as *Alistipes onderdonki*, *Bacteroides dorei*, and *Parabacteroides merdae*. *Alistipes onderdonki* was found in the stool of patients with multiple system atrophy, suggesting its role in α-synucleinopathy^49^. *Bacteroides dorei* was associated with weight loss and autoimmune disease, and *Parabacteroides merdae* was enriched in colorectal cancer^50^. In addition, we found a significant species-level difference in the *Prevotella* genus between PD patients and HCs using shotgun sequencing. There are more than 40 species in this genus^51^. In previous studies, Scheperjans et al. found that the *Prevotellaceae* family was decreased in PD^52^ and Petrov et al. found that *Prevotella copri* was decreased in PD^53^. On the other hand, Wallen et al. reported that the *Prevotella_9* genus, including *Prevotella copri*, was decreased in PD, but the less common genus *Prevotella* was increased in PD^54^. Therefore, the analysis accuracy depends on the phylogenetic resolution. We found that *Prevotella copri* was less abundant in PD patients than in HCs and it was associated with decreased arginine and glutamate metabolism. To date, little is known about whether *Prevotella copri* significantly affects the pathogenesis of PD. *Prevotella* is usually abundant in the stool of healthy Asian people, and it benefits the host by helping to digest a high-fiber diet^55^. The role of *Prevotella copri* in PD in association with amino acid metabolism requires further investigation, especially in Asian populations.

By directly comparing the discriminatory ability of shotgun and 16 S rRNA gene sequencing, we found that shotgun metagenomic sequencing performs better at discriminating PD patients from HCs. Previous studies have investigated the gut microbiome in PD patients using 16 S rRNA gene sequencing^25^, and only a small number of recent studies have used shotgun metagenomic sequencing for PD^24,56^. One study found a mean AUC of 0.92 when discriminating PD patients from HCs using gut metagenomics-derived gene markers^24^, which is comparable to our maximum AUC of 0.88. We found that gene markers and functional pathways determined using shotgun sequencing had better discriminatory performance than the taxonomic composition, which might be explained by the high levels of functional redundancy in the microbiota^23,38,39^. In addition, the taxonomic composition derived from shotgun sequencing showed better discriminatory performance than the taxonomic composition derived from 16 S rRNA gene sequencing. These results support the superiority of shotgun metagenomic sequencing over 16 S rRNA gene sequencing for identifying relevant changes to the PD-associated gut microbiota.

The present study has some limitations. First, although our 16 S rRNA gene data revealed significant differences in the oral microbiome between PD patients and HCs, shotgun metagenomic sequencing was performed only on stool samples. Because the gut microbiome showed better discriminating performance for PD than the oral microbiome, whole-genome shotgun metagenomic sequencing was performed on the gut microbiome, considering the cost-effectiveness. Further studies using shotgun metagenomics are therefore required to identify the oral microbial functions and species-level composition. Second, we did not investigate the metabolites associated with the identified gut and oral microbiota. To support the results of our functional analysis on the significant alterations in glutamate and arginine metabolism, these metabolites should be investigated in stool samples.

In conclusion, the present study identified a distinctive connection between the oral and gut microbiota, which might lead to functional alterations of the PD-associated microbiome.

## Methods

### Study participants

In this case-control study, we prospectively enrolled patients with PD using the UK PD Society brain bank clinical diagnostic criteria^57^ and their spouses as HCs at Asan Medical Center from 2019 to 2020. The patients’ spouses were selected as HCs because they share common environmental factors. The exclusion criteria were as follows: (1) participants with inflammatory bowel diseases; (2) participants with a history of acute inflammatory or infectious disease within one month prior to participation; (3) participants using antibiotics, steroids, or immunosuppressants; (4) participants who underwent surgery on their gastrointestinal tracts or oral cavity; (5) participants using artificial nutrition; (6) participants who had undergone deep brain stimulation; and (7) participants diagnosed with PD dementia.

### Ethics

This study was approved by the Asan Medical Center Institutional Review Board (2019-0929) and was performed in accordance with the relevant guidelines and regulations, including the Declaration of Helsinki. All participants provided written informed consent at study enrollment.

### Clinical evaluation

We assessed the baseline characteristics of the cohort, including age, sex, and body mass index. Diet was assessed using a semi-quantitative food frequency questionnaire^58^. Irritable bowel syndrome and constipation were assessed using the ROME III diagnostic criteria^59^. Dysphagia was assessed using a swallowing disturbance questionnaire^60^, and olfactory function was assessed using a scent survey for screening (SSS) test^61^. Motor function was assessed using the Unified PD Rating Scale (UPDRS) and Hoehn and Yahr (H&Y) stage in the medication-off state.

### Preparation of oral and stool samples

Oral swabs and stool samples of patients with PD and their spouses were collected for microbial community analysis. For the oral swab samples, the buccal area was swabbed with an eSwab kit (COPAN Diagnostics Inc., California, USA). A stool sampling kit (CJ Bioscience Inc., Seoul, Korea) was used to collect stool samples. Conventional 16 S rRNA gene sequencing was performed for both the oral and gut microbiome. Because we found that the gut microbiome showed better performance when discriminating PD from controls than the oral microbiome, whole-genome shotgun metagenomic sequencing was only performed on the gut microbiome, considering the cost-effectiveness.

### 16 S rRNA gene sequencing, taxonomic profiling, and functional profiling

The V3-4 hypervariable region of the 16 S rRNA gene was amplified with primers 341 F and 805 R using the direct PCR method. Libraries were prepared using an NEBNext Ultra II FS DNA Library Prep Kit for Illumina (New England Biolabs, Ipswich, MA, USA). The prepared DNA libraries were sequenced by CJ Bioscience Inc. (Seoul, Korea) using the Illumina Miseq platform (Illumina, San Diego, CA, USA) with 2 × 300 bp kits.

The paired end raw 16 S rRNA sequences data were uploaded to the EzBioCloud and processed using a web-based EzBioCloud microbiome taxonomic profile tool (https://www.ezbiocloud.net/contents/16smtp). High-quality sequence reads were assigned to “species group” at 97% sequence similarity using the PKSSU4.0 database. The prediction of functional biomarkers of the oral microbiota was performed using the PICRUSt with EzBioCloud MTP server^62^.

### Whole-genome shotgun metagenomic sequencing

Whole-genome shotgun metagenomic libraries were prepared using the NEBNext Ultra II DNA Library Prep Kit and the NEBNext Multiplex Oligos for Illumina (New England Biolabs, Ipswitch, USA), according to the manufacturer’s protocols. Fragment size and DNA concentration in the final library were checked using a Bioanalyzer system (Agilent Technologies, Santa Clara, USA) before sequencing using an Illumina NovaSeq 6000 platform (2×150 bp read length) at Macrogen (Seoul, Korea).

### Taxonomic profiling of shotgun metagenomics data

A Kraken2 database^63^ containing bacterial and archaeal species represented in the EzBioCloud database was generated^64^. For each species, 92 core genes were extracted using the UBCG pipeline^65^. The total core gene length for each species was stored for further downstream analysis. A Kraken2-compatible taxonomic structure was constructed using EzBioCloud’s taxonomic system, and the core gene sequences were converted into FASTA files using a numerical identifier matching the taxonomic structure file. Finally, the database was compiled with the Kraken2-build command using a k of size 35 and default parameters.

The potential presence of bacterial and archaeal species for each raw metagenomic sample read was initially surveyed using the pre-built Kraken2 core gene database^66^. After acquiring a list of candidate species, a custom Bowtie2 database was built, utilizing only the core genes from the species found during the first step to reduce the search space and obtain accurate coverage and depth metrics. The raw sample was then mapped against the Bowtie2 database using the very sensitive option and a quality threshold of phred33. Samtools was used to convert and sort the output BAM file. Coverage of the mapped reads against the BAM file was obtained using Bedtools. To avoid false positives, reads that mapped to a given species were only quantified if the total coverage of their core genes was at least 25% according to an in-house script. Finally, species abundance was calculated using the total number of reads counted, and normalized species abundance was calculated using the total length of core genes per species.

### Functional profiling based on shotgun metagenomics data

Functional annotations were obtained by matching each read against the KEGG database^67^ using DIAMOND^68^. An initial database file was built from the KEGG fasta file containing the ortholog amino acid sequences using DIAMOND’s makedb command with the default parameters. Then, DIAMOND was executed using the blastx parameter, which converts each metagenomic read into multiple amino acid sequences by generating all six open reading frames and then matching these against the pre-built KEGG database. If a read had multiple KEGG hits, the top hit was used. After quantifying all of the KEGG orthologs, minpath was used to predict the presence of KEGG functional pathways^69^.

### Machine learning for discriminating PD

Using the 16 S rRNA-based sequencing data for the oral and gut microbiome and the whole-genome shotgun sequencing data for the gut microbiome, we developed a random forest classifier for discriminating PD from HCs, using custom python scripts employing the Scikit-learn package^70^. We trained random forest classifiers with the bacterial composition (oral = genus level; gut = species and genus level) and function (gene and pathway level). The model was trained 20 times using a 5-fold cross-validation method, and the average area under the receiver operating characteristic (ROC) curve was calculated.

### Statistical analysis

We compared the baseline demographics, dietary intake, and clinical symptoms between PD patients and the HCs using a χ^2^ test, Student’s *t*-test, and Mann–Whitney U test, where appropriate. Significance was set at a *P* value less than 0.05, and all *P* values were 2-tailed. The species richness was assessed using Chao1, and diversity indices were calculated using the Shannon matrix. The beta-diversity was calculated using the Bray-Curtis metric. The significance of beta-diversity was assessed using PERMANOVA with QIIME2^71^. In the diversity analyses, all features were used.

To examine the taxonomic and functional differences between PD and HC, we performed LEfSe analysis^72^, which uses effect size to measure phenotypic differences in metagenomic data, as well as statistical significance. Features with less than 0.01% relative abundance in the data were excluded from the analysis to avoid obtaining biologically meaningless results. In addition, we performed beta-diversity and LEfSe analyses to compare the microbiota between patients whose H&Y stage was less than 3 (mild PD) and patients whose H&Y stage was 3 or more (severe PD).

The variation in each taxonomic profile and function between PD patients and HCs was analyzed using a Mann–Whitney U test. The Benjamini–Hochberg method was used to adjust for multiple testing. Statistical significance was set at an adjusted *P* value (*Q*-value) of 0.05. Since the oral microbiome could affect the gut microbiome^15^, the correlation between the oral and gut microbiome composition was determined using linear regression analysis. We imposed a centered log-ratio transformation on the relative abundance data using the ‘phyloseq’ and ‘microbiome’ R packages^73–75^.

Canonical correspondence analysis (CCA) was performed to identify the bacterial taxa associated with the clinical symptoms of PD. In CCA, we used three demographic features (age, BMI, and disease duration), three drugs that can affect the microbiota (COMT inhibitor, amantadine, and dopamine agonist), and the clinical symptoms of PD (Bristol stool scale indicating stool firmness, IBS symptoms, dysphagia scale, olfaction, H&Y stage and UPDRS). The top 20 strains from LEfSe analysis were used in the analysis. CCA was performed using XLSTAT software (Addinsoft, Paris). Linear regression analysis was also conducted to highlight the clinically meaningful and statistically significant correlation from the CCA.

Network analysis was performed using the top 20 pathway level functions and the top 20 species level taxa based on the LDA Score. Network maps were generated between bacterial species and functional pathways using QIIME2 SCNIC and visualized in Cytoscape version 3.8.2^76^. Hierarchical all-against-all association testing was performed to find multi-resolution associations between the bacterial taxonomic and functional profiles^77^.

## Supplementary information

Supplementary materials

## Data Availability

The datasets generated during the current study are available in the NCBI repository (https://www.ncbi.nlm.nih.gov) and Sequence Read Archive database under the accession numbers PRJNA742875 and PRJNA743718, respectively. PRJNA742875 contains 16S rRNA gene sequencing data from 356 samples, including 172 stool samples (88 PD and 84 HC), 143 oral samples (74 PD, 69 HC), and 41 nasal samples. PRJNA743718 contains shotgun metagenomic sequencing data from 156 stool samples, reflecting the dataset after quality control filtering. The associated metadata include group identifiers in the “host_status” column, indicating whether each sample is from a Parkinson’s Disease (PD) patient or a healthy control (HC).

## Code availability

No custom codes were used. All software and packages, their versions, relevant specification and parameters are stated in the Methods section.
