# Supplementary material for: Oral and gut dysbiosis leads to functional alterations in Parkinson’s disease
Source: NPJ Parkinsons Dis. 2022 Jul 7;8:87. doi: 10.1038/s41531-022-00351-6 (PMC9262988; doi:10.1038/s41531-022-00351-6)
Supplement: Supplementary file 1 — Supplementary materials [file 41531_2022_351_MOESM1_ESM.pdf]

Supplementary materials

Sungyang Jo, MD[1,†]; Woorim Kang, BS[2,3,†]; Yun Su Hwang, MD[1]; Seung Hyun Lee, MD[1]; Kye Won Park, MD[4]; Ja Yeon Do, BS[1]; Mi Sun Kim, RN, PhD[1]; Hyunna Lee, PhD[5]; Hyung Jeong Yoon, BS[6]; Yoo Kyung Park, PhD[6]; Mauricio Chalita, PhD[3]; Je Hee Lee, PhD[3]; Hojun Sung, BS[2]; Jae-Yun Lee, BS[2]; Jin-Woo Bae, PhD[2]; Sun Ju Chung MD, PhD[1]

[†]**These authors equally contributed to the manuscript**

Supplementary Figure 1. Study flowchart

Supplementary Figure 2. Comparison of alpha diversity indices between patients with Parkinson's disease (PD) and healthy controls (a) in the stool and (b) in oral samples using 16S rRNA sequencing

Supplementary Figure 3. Gut and oral microbiota associated with disease severity in Parkinson's disease (PD) using 16s rRNA sequencing.

Supplementary Figure 4. Correlation between clinical symptoms and gut microbial abundance

Supplementary Figure 5. Glutamate and arginine synthesis-associated pathway maps

Supplementary Figure 6. Variable importance of features in random forest classification models

Supplementary Table 1. Baseline clinical characteristics of patients with Parkinson's disease (PD) and healthy controls with complete stool sequencing data.

Supplementary Table 2. Baseline clinical characteristics of patients with Parkinson's disease (PD) and healthy controls with complete oral sequencing data.

Supplementary Table 3. Functional biomarkers of the oral microbiome using PICRUSt

Supplementary Table 4. Correlation between clinical symptoms and gut microbial abundance

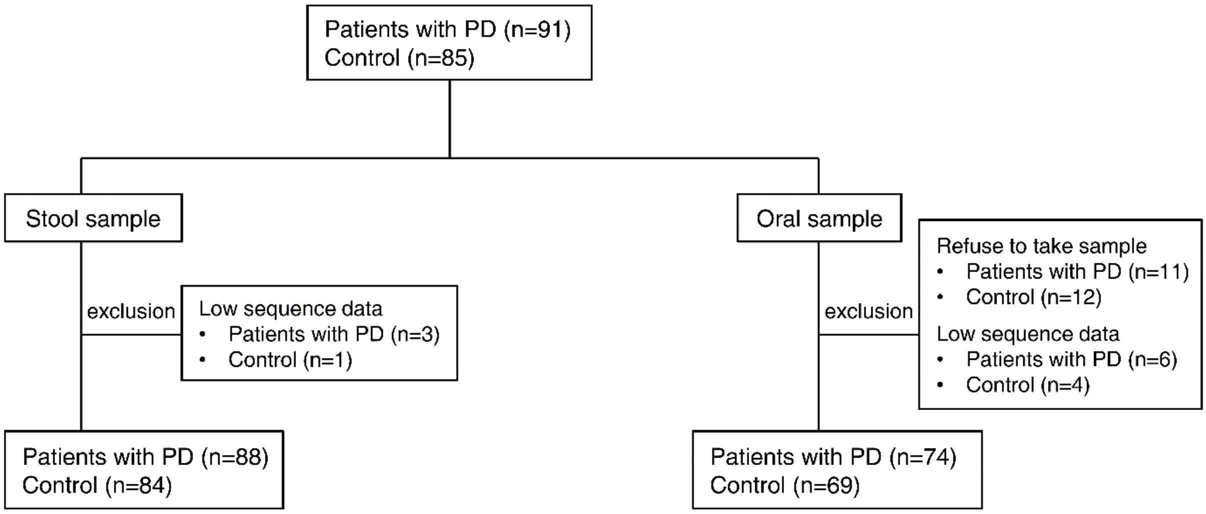

Supplementary Figure 1. Study flowchart.

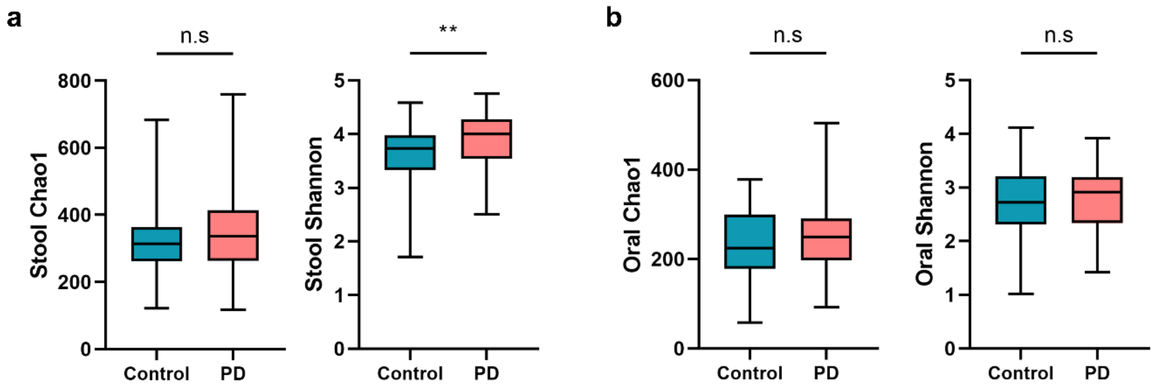

Supplementary Figure 2. Comparison of alpha diversity indices between patients with Parkinson's disease (PD) and healthy controls (a) in the stool and (b) in oral samples using 16S rRNA sequencing Boxplot centerline represents the median (50th percentile). The top and bottom hinges represent 75th and 25th percentiles, respectively. The upper and lower whiskers correspond to the highest and lowest data points. n.s : not significant, $*P < 0.05$, $**P < 0.01$

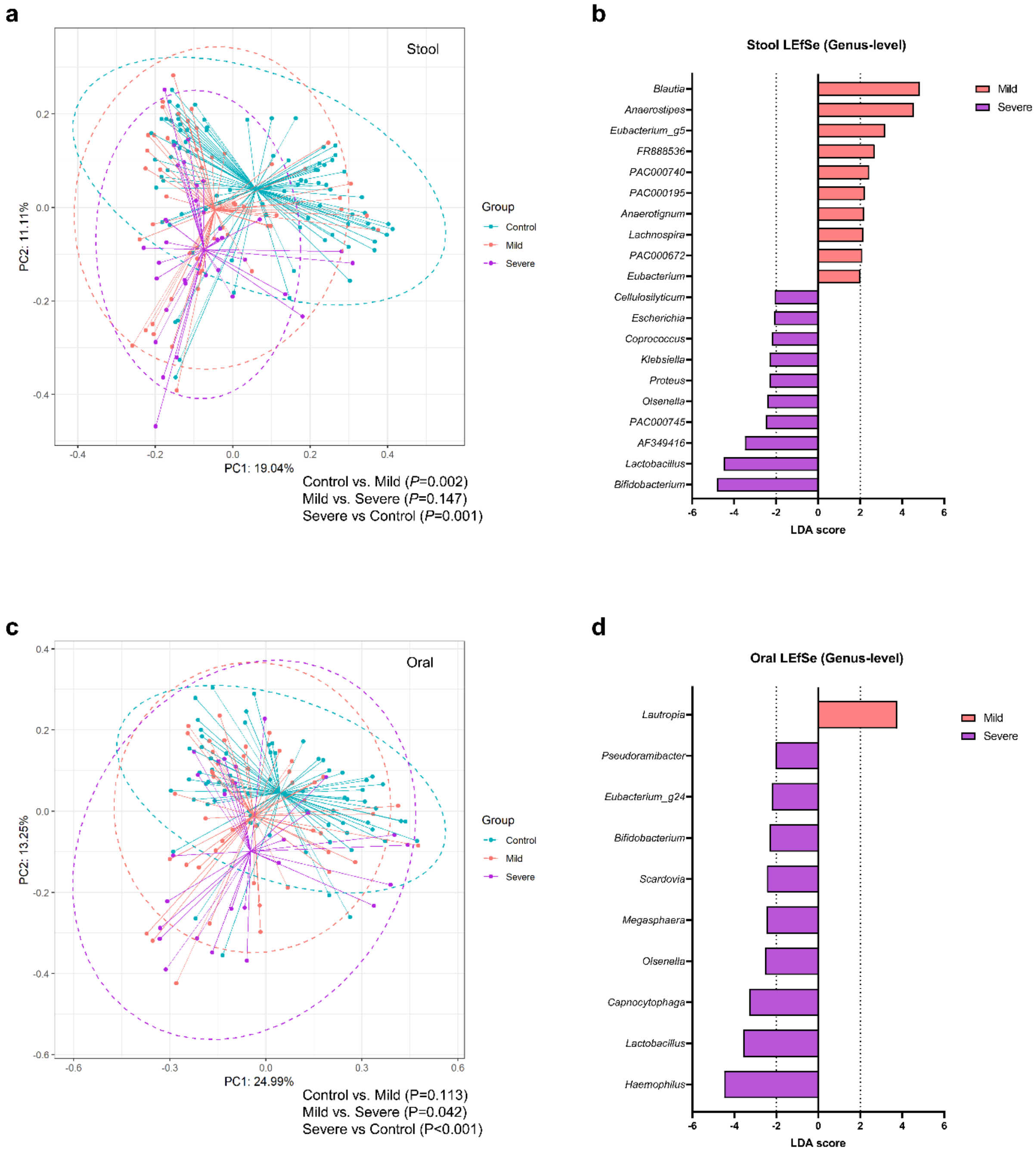

Supplementary Figure 3. Gut and oral microbiota associated with disease severity in Parkinson's disease (PD) using 16s rRNA sequencing. (a) Comparison of beta-diversity between mild PD, severe PD, and healthy controls for gut microbiome (b) Comparison of gut microbiome between mild PD and severe PD using LEfSe analysis (c) Comparison of beta-diversity between mild PD, severe PD, and healthy controls for oral microbiome (d) Comparison of oral microbiome between mild PD and severe PD using LEfSe analysis

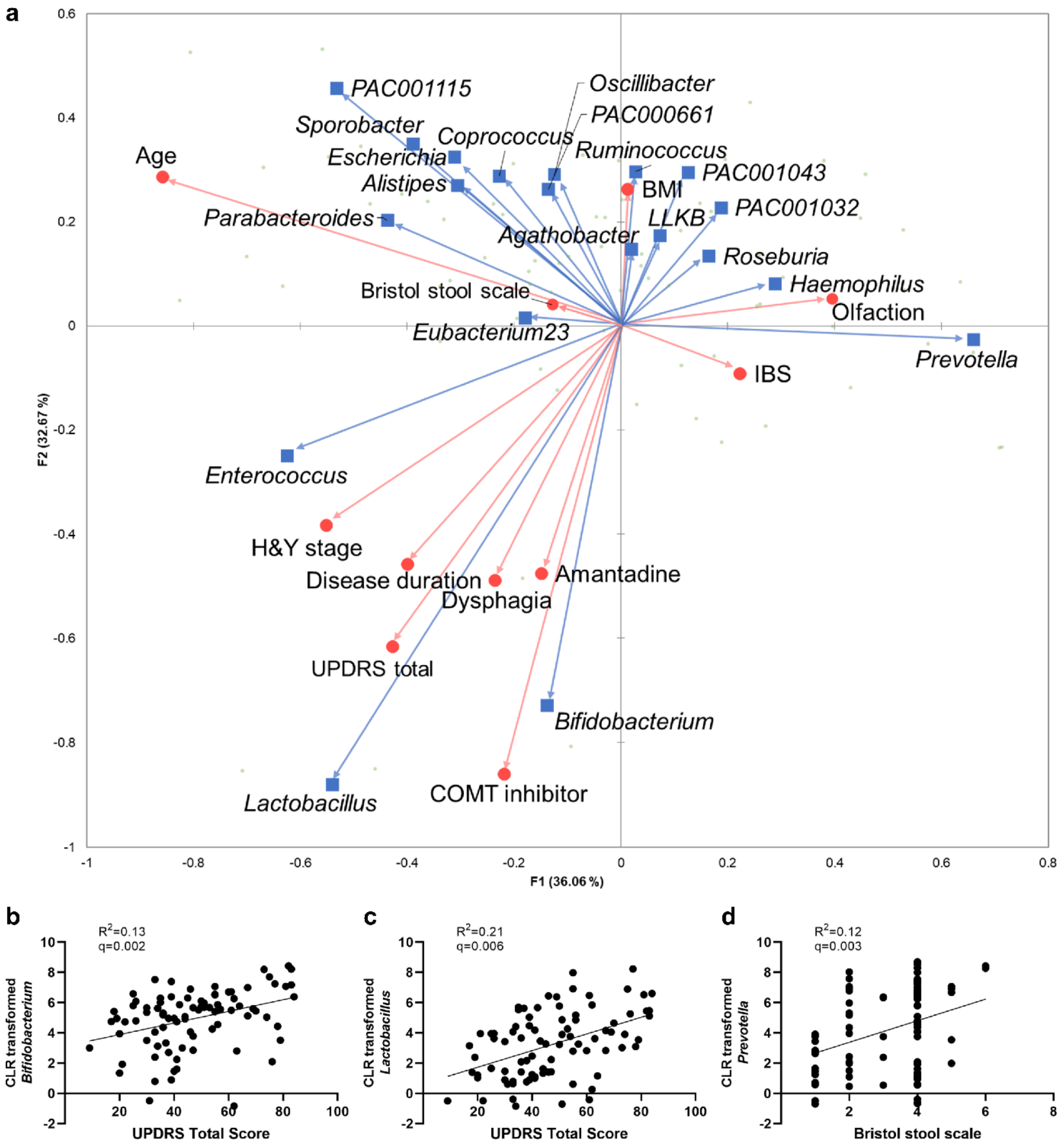

Supplementary Figure 4. Correlation between clinical symptoms and gut microbial abundance. (a) Canonical correlation analysis between Parkinson's disease-related clinical features and gut microbial abundance. (b-c) linear regression analysis between UPDRS and (b) stool *Bifidobacterium*, and (c) stool *Lactobacillus* (d) linear regression analysis between stool firmness and stool *Prevotella*.

a

Glutamate and GABA biosynthesis (from ko00250, k00910)

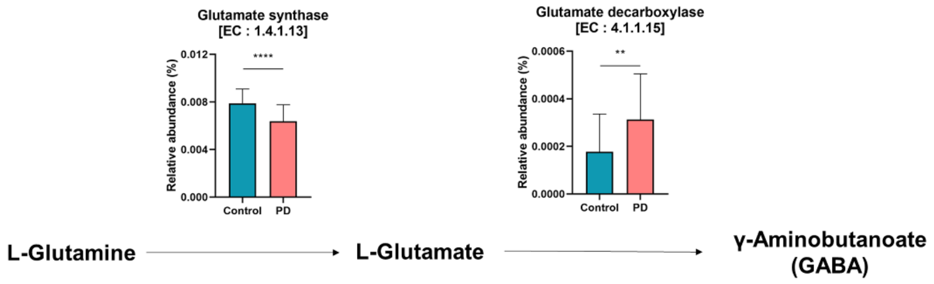

b

Glutamate biosynthesis from carbohydrates (from ko00500, ko000010, ko00020, ko00020, 00250, ko00910)

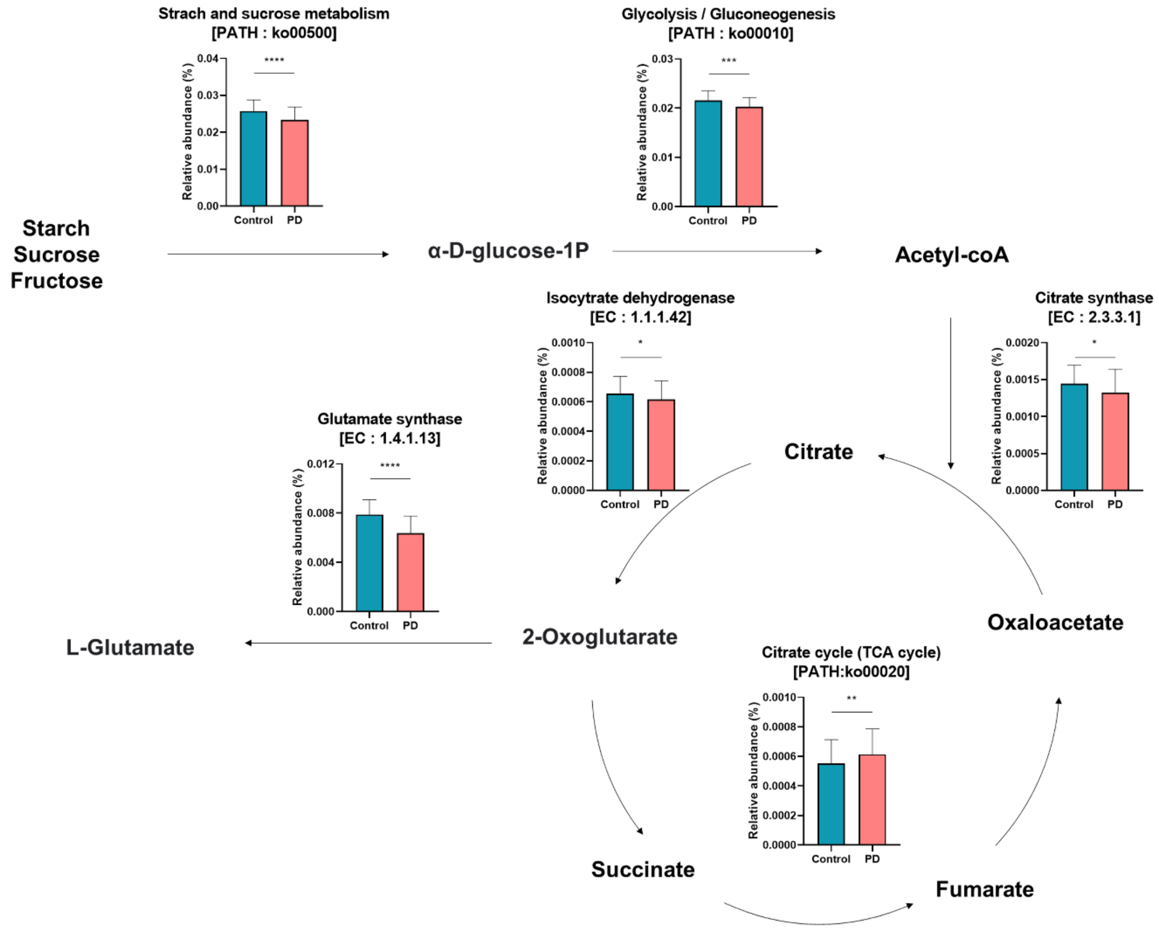

c

Glutamate biosynthesis during purine metabolism (from ko00250)

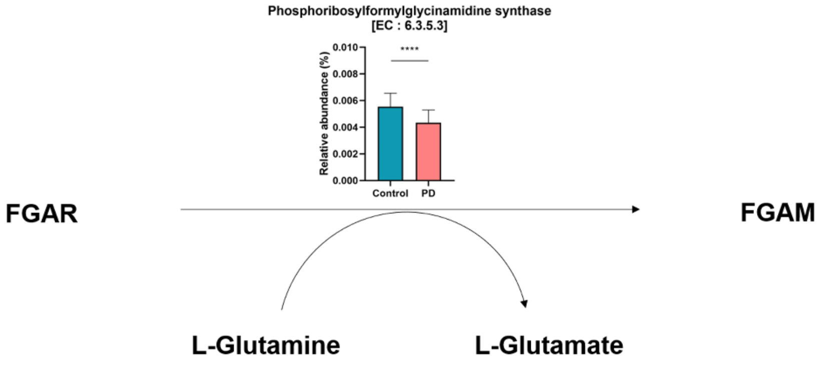

d

Arginine biosynthesis (from ko00220, ko00910)

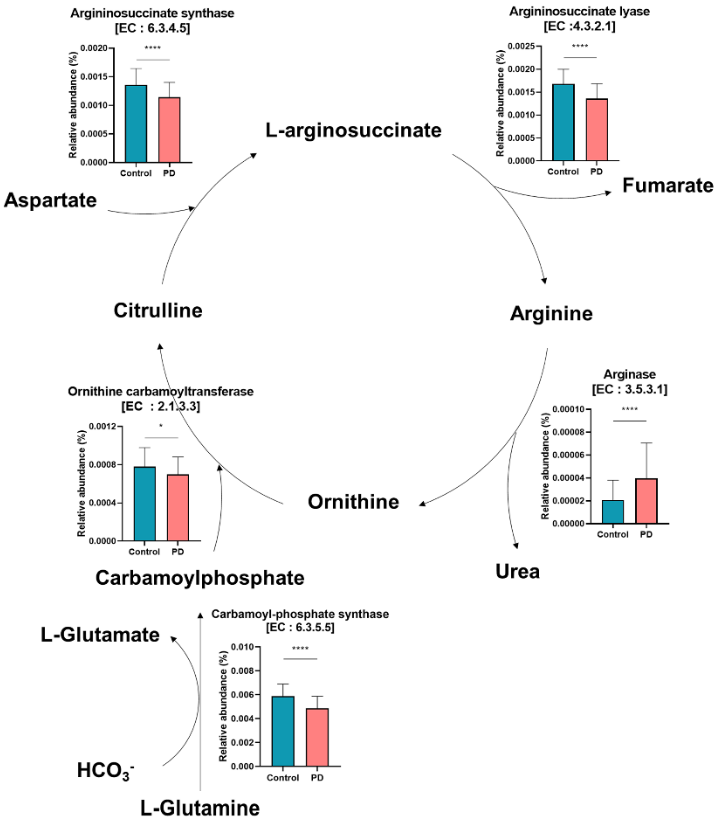

Supplementary Figure 5. Glutamate and arginine synthesis-associated pathway maps. Bar plots indicate mean abundance of genes and error bars represent standard deviation, *$P < 0.05$, **$P < 0.01$, ***$P < 0.001$, ****$P < 0.0001$.

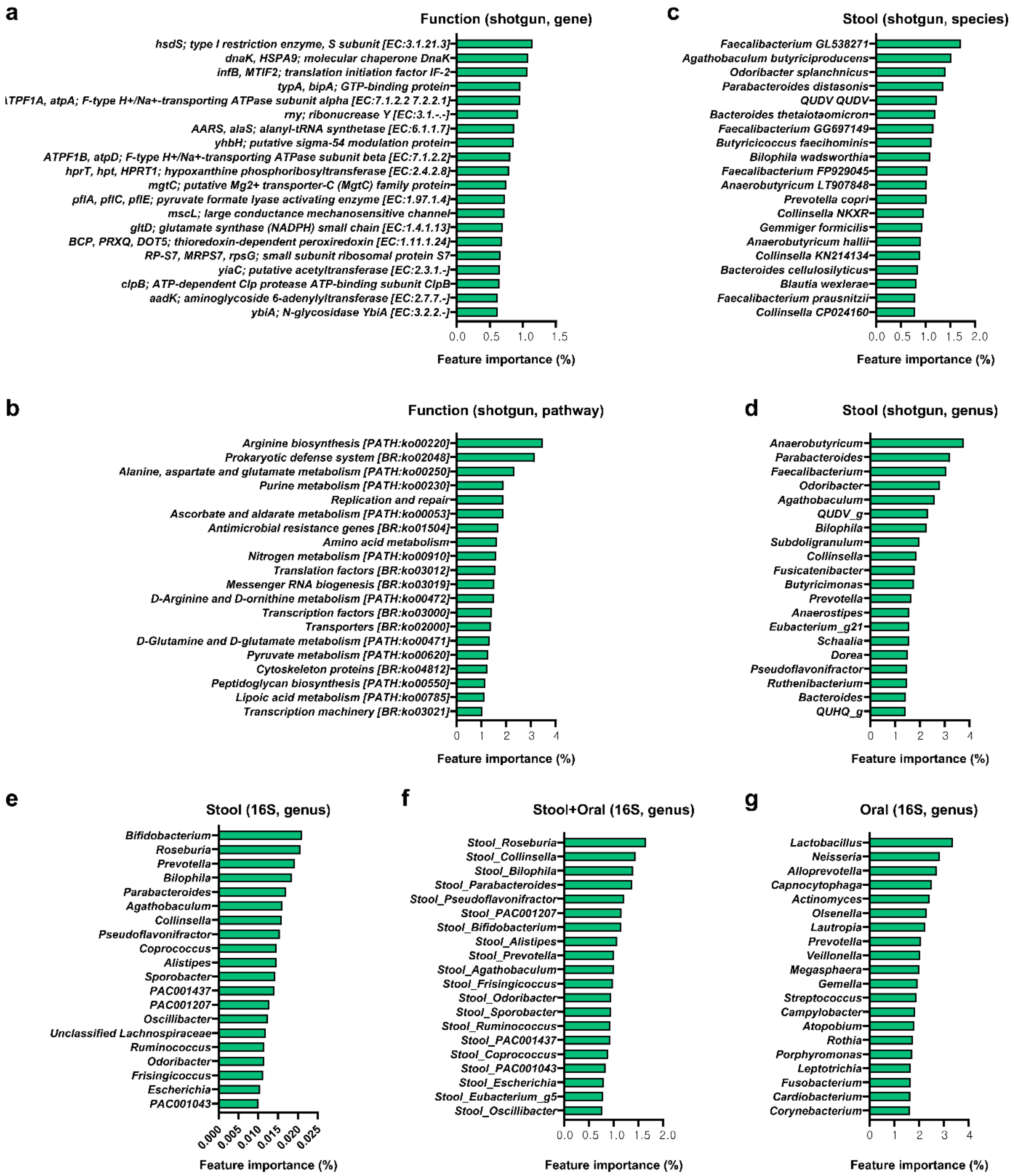

Supplementary Figure 6. Variable importance of features in random forest classification models.

Supplementary Table 1. Baseline clinical characteristics of patients with Parkinson's disease (PD) and healthy controls with complete stool sequencing data.

	**PD (n=88)**	**Controls (n=84)**	***p* value**
Age, years	65.1 ± 7.8	64.7 ± 8.1	0.734
Male, n (%)	47 (53.4%)	39 (46.4%)	0.446
BMI, kg/m^2	24.3 (22.2–26.2)	23.9 (21.5–25.4)	0.106
Education, years	12.0 (12.0–16.0)	12.0 (12.0–16.0)	0.980
Clinical symptoms			
IBS, n (%)	3 (3.4%)	3 (3.6%)	1.000
Constipation, n (%)	41 (46.6%)	10 (11.9%)	<0.001
Bristol	4.0 (2.0– 4.0)	4.0 (4.0– 4.0)	<0.001
Swallowing test	0.0 (0.0– 3.0)	0.0 (0.0– 0.0)	0.003
Smell test	9.0 (6.0–10.0)	10.0 (10.0–10.0)	<0.001
Disease duration	2.5 (0.0– 6.5)	-	
UPDRS			
UPDRS part 1	5.0 (3.0– 7.0)	-	
UPDRS part 2	7.0 (4.0–11.0)		
UPDRS part 3	32.0 (25.0–40.0)		
UPDRS part 4	0.0 (0.0– 2.0)		
UPDRS total score	45.0 (34.5–60.5)		
Hoehn and Yahr stage	2.0 (2.0– 3.0)		
Medication for PD			
Levodopa use, n (%)	73 (83.0%)	-	

Dopamine agonist, n (%)	34 (38.6%)	-	
COMT inhibitor, n (%)	7 (8.0%)	-	
MAO-B inhibitor, n (%)	25 (28.4%)	-	
Amantadine, n (%)	19 (21.6%)	-	
Levodopa equivalent daily dose (mg)	455.0 (300.0−822.2)	-	
Daily dietary intake	(n=80)	(n=77)	
Total energy (kCal)	2183.0 ± 503.8	2154.8 ± 568.2	0.743
Carbohydrate (g)	297.7 ± 68.8	287.9 ± 80.0	0.415
Protein (g)	88.9 ± 26.0	88.8 ± 25.3	0.975
- animal source	47.9 ± 15.3	47.1 ± 16.6	0.758
- plant source	41.0 ± 16.7	41.7 ± 16.4	0.806
Fat intake (g)	69.5 ± 26.3	67.7 ± 24.3	0.665
- animal source	41.0 ± 19.8	38.8 ± 18.5	0.482
- plant source	28.5 ± 13.1	28.9 ± 12.9	0.847
Fiber (g)	39.0 ± 17.0	38.0 ± 17.7	0.727
- Soluble fiber	5.1 ± 2.5	5.1 ± 2.4	0.959
- Insoluble fiber	19.1 ± 8.1	19.2 ± 8.6	0.944

Supplementary Table 2. Baseline clinical characteristics of patients with Parkinson's disease (PD) and healthy controls with complete oral sequencing data.

	**PD (n=74)**	**Controls (n=69)**	***p* value**
Age, years	65.0 ± 7.6	64.9 ± 7.5	0.911
Male, n (%)	42 (56.8%)	33 (47.8%)	0.368
BMI, kg/m^2	24.3 (22.3−26.3)	24.1 (22.0−25.7)	0.492
Education, years	12.0 (12.0−16.0)	12.0 (10.5−16.0)	0.915
Clinical symptoms			
IBS, n (%)	2 (2.7%)	2 (2.9%)	1.000
Constipation, n (%)	34 (45.9%)	8 (11.6%)	<0.001
Bristol	4.0 (2.0− 4.0)	4.0 (4.0− 4.0)	<0.001
Swallowing test	0.0 (0.0− 3.0)	0.0 (0.0− 0.0)	0.008
Smell test	9.5 (6.0−10.0)	10.0 (10.0−10.0)	<0.001
Disease duration	2.0 (0.0− 6.0)	-	
UPDRS			
UPDRS part 1	5.0 (3.0− 8.0)	-	
UPDRS part 2	7.0 (3.0−11.0)		
UPDRS part 3	32.0 (23.0−41.0)		
UPDRS part 4	0.0 (0.0− 0.0)		
UPDRS total score	43.0 (34.0−61.0)		
Hoehn and Yahr stage	2.0 (2.0− 3.0)		
Medication for PD			
Levodopa use, n (%)	59 (79.7%)	-	
Dopamine agonist, n (%)	28 (37.8%)	-	

COMT inhibitor, n (%)	4 (5.4%)	-	
MAO-B inhibitor, n (%)	21 (28.4%)	-	
Amantadine, n (%)	12 (16.2%)	-	
Levodopa equivalent daily dose (mg)	450.0 (200.0−712.5)	-	
Daily dietary intake	(n=68)	(n=64)	
Total energy (kCal)	2184.8 ± 501.5	2230.7 ± 571.6	0.624
Carbohydrate (g)	296.9 ± 72.5	297.2 ± 79.6	0.980
Protein (g)	89.7 ± 26.2	91.4 ± 25.6	0.707
- animal source	47.5 ± 15.9	48.6 ± 17.1	0.702
- plant source	42.2 ± 16.7	42.8 ± 16.5	0.836
Fat intake (g)	68.5 ± 26.2	69.6 ± 24.8	0.803
- animal source	39.8 ± 20.2	40.3 ± 19.4	0.889
- plant source	28.7 ± 12.4	29.3 ± 12.7	0.774
Fiber (g)	38.9 ± 17.1	39.6 ± 18.6	0.806
- Soluble fiber	5.2 ± 2.5	5.3 ± 2.5	0.733
- Insoluble fiber	19.0 ± 8.1	19.9 ± 9.0	0.575

Supplementary Table 3. Functional biomarkers of the oral microbiome using PICRUSt

Ortholog	Definition	Pathway	Module	p-value	FDR adjusted q-value	PD	Control
K03488	beta-glucoside operon transcriptional antiterminator			0.000208	0.234752	0.0316	0.0252
K02199	cytochrome c biogenesis protein CcmG, thiol:disulfide interchange protein DsbE			0.000304	0.234752	0.0447	0.0533
K15634	probable phosphoglycerate mutase	ko00010, ko00260, ko00680, ko01100, ko01110, ko01120, ko01130, ko01200, ko01230	M00001, M00002, M00003	0.000569	0.234752	0.0326	0.0225
K06188	aquaporin Z			0.000613	0.234752	0.0239	0.0301
K06962	uncharacterized protein			0.000641	0.234752	0.0211	0.0159
K03546	DNA repair protein SbcC/Rad50			0.000811	0.234752	0.0406	0.035
K00689	dextransucrase	ko00500, ko02020		0.000847	0.234752	0.0661	0.0371
K07493	putative transposase			0.000859	0.234752	0.0491	0.0325
K15539	cytoskeleton protein RodZ			0.001022	0.234752	0.0344	0.0407
K02065	phospholipid/cholesterol/gamma-HCH transport system ATP-binding protein	ko02010	M00210, M00669, M00670	0.001022	0.234752	0.0651	0.0733
K08978	bacterial/archaeal transporter family protein			0.001582	0.234752	0.0401	0.0513
K19225	rhomboid protease GluP			0.001998	0.234752	0.0338	0.0277
K05988	dextranase	ko00500		0.002025	0.234752	0.0134	0.0078

K13732	fibronectin-binding protein A	ko05100		0.002546	0.234752	0.0149	0.0092
K21125	mucin-5AC	ko04657		0.002869	0.234752	0.0235	0.0166
K06148	ATP-binding cassette, subfamily C, bacterial			0.003105	0.234752	0.0719	0.063
K19736	TetR/AcrR family transcriptional regulator, regulator of autoinduction and epiphytic fitness			0.003676	0.236216	0.0224	0.0171
K03646	colicin import membrane protein			0.003871	0.236216	0.0687	0.074
K06196	cytochrome c-type biogenesis protein			0.003871	0.236216	0.0902	0.102
K09765	epoxyqueuosine reductase			0.004074	0.236216	0.0308	0.0369
K17320	putative aldouronate transport system permease protein	ko02010	M00603	0.004127	0.236216	0.0203	0.026
K02477	two-component system, LytTR family, response regulator			0.004745	0.241451	0.0284	0.0235
K17318	putative aldouronate transport system substrate-binding protein	ko02010	M00603	0.004805	0.241451	0.0211	0.0273
K12541	ATP-binding cassette, subfamily C, bacterial LapB	ko02010	M00330	0.005052	0.241852	0.0217	0.0281
K07029	diacylglycerol kinase (ATP)	ko00561, ko00564, ko01100, ko01110		0.005052	0.241852	0.0413	0.0341
K00788	thiamine-phosphate pyrophosphorylase	ko00730, ko01100	M00127	0.005513	0.244934	0.0538	0.059
K06950	uncharacterized protein			0.005513	0.244934	0.0345	0.0278
K12373	hexosaminidase	ko00511, ko00513, ko00520, ko00531, ko00603, ko00604, ko01100,	M00079	0.006238	0.259034	0.0684	0.0826

		ko04142					
K16554	polysaccharide biosynthesis transport protein	ko05111		0.006472	0.261413	0.0366	0.0417
K03547	DNA repair protein SbcD/Mre11			0.006795	0.262681	0.0357	0.0299
K15923	alpha-L-fucosidase 2	ko00511		0.007577	0.273064	0.0186	0.0242
K03308	neurotransmitter:Na+ symporter, NSS family			0.007577	0.273064	0.0551	0.0624
K19334	biofilm protein TabA			0.00824	0.277262	0.0276	0.0331
K07718	two-component system, sensor histidine kinase YesM	ko02020	M00519	0.00824	0.277262	0.045	0.0556
K01208	cyclomaltodextrinase / maltogenic alpha-amylase / neopullulanase	ko00500, ko01100		0.008539	0.277262	0.0235	0.0291
K07494	putative transposase			0.008641	0.279278	0.029	0.0378
K01286	D-alanyl-D-alanine carboxypeptidase			0.008744	0.280666	0.0296	0.0221
K12960	5-methylthioadenosine/S-adenosylhomocysteine deaminase	ko00270, ko01100		0.008849	0.28079	0.0494	0.0619
K01436	amidohydrolase			0.008849	0.28079	0.0514	0.0428
K06199	fluoride exporter			0.009498	0.285211	0.0686	0.076
K16692	tyrosine-protein kinase Etk/Wzc			0.009723	0.286882	0.0226	0.017
K16087	hemoglobin/transferrin/lactoferrin receptor protein			0.012255	0.286882	0.0362	0.0496
K01448	N-acetylmuramoyl-L-alanine amidase	ko01503	M00727	0.013727	0.288501	0.0802	0.066
K07238	zinc transporter, ZIP family			0.015354	0.296986	0.0265	0.0201
K12996	rhamnosyltransferase			0.015525	0.298433	0.0282	0.0226
K01190	beta-galactosidase	ko00052, ko00511, ko00600, ko01100		0.01696	0.304242	0.0687	0.0786
K02761	PTS system, cellobiose-specific IIC component	ko00500, ko02060	M00275	0.019753	0.319929	0.0292	0.0231
K07025	putative hydrolase of the HAD superfamily			0.020621	0.324901	0.0848	0.0761

K03673	thiol:disulfide interchange protein DsbA	ko01503	M00728	0.021522	0.324901	0.0152	0.0197
K06147	ATP-binding cassette, subfamily B, bacterial			0.021522	0.324901	0.3676	0.341
K01788	N-acylglucosamine-6-phosphate 2-epimerase	ko00520		0.021753	0.326271	0.0516	0.0602
K01186	sialidase-1	ko00511, ko00600, ko04142		0.022221	0.328618	0.0575	0.0712
K17624	endo-alpha-N-acetylgalactosaminidase			0.022458	0.328618	0.027	0.0336
K19302	undecaprenyl-diphosphatase	ko00550		0.022458	0.328618	0.0665	0.0613
K00275	pyridoxamine 5'-phosphate oxidase	ko00750, ko01100, ko01120	M00124	0.022939	0.332302	0.0155	0.0202
K01081	5'-nucleotidase	ko00230, ko00240, ko00760, ko01100, ko01110		0.023428	0.334548	0.059	0.0476
K03707	thiaminase (transcriptional activator TenA)	ko00730, ko01100		0.025215	0.345598	0.0335	0.0396
K20344	ATP-binding cassette, subfamily C, bacteriocin exporter	ko02010, ko02024		0.025215	0.345598	0.0252	0.0168
K12267	peptide methionine sulfoxide reductase msrA/msrB			0.026288	0.351448	0.0733	0.0798
K01756	adenylosuccinate lyase	ko00230, ko00250, ko01100, ko01110, ko01130	M00048, M00049	0.026288	0.351448	0.0961	0.1061
K00812	aspartate aminotransferase	ko00220,		0.027971	0.362763	0.0604	0.0548

		ko00250, ko00270, ko00330, ko00350, ko00360, ko00400, ko00401, ko00950, ko00960, ko01100, ko01110, ko01130, ko01210, ko01230					
K01696	tryptophan synthase beta chain	ko00260, ko00400, ko01100, ko01110, ko01130, ko01230	M00023	0.029144	0.36643	0.0667	0.0722
K00797	spermidine synthase	ko00270, ko00330, ko00410, ko00480, ko01100	M00034, M00133	0.031617	0.384739	0.0601	0.0664
K01090	protein phosphatase			0.031939	0.386968	0.0415	0.0475
K03892	ArsR family transcriptional regulator, arsenate/arsenite/antimonite-responsive transcriptional repressor			0.033253	0.393022	0.0452	0.0401
K08659	dipeptidase			0.034268	0.400293	0.0338	0.0262
K07486	transposase			0.03531	0.405694	0.0329	0.0271
K07707	two-component system, LytTR	ko02020,	M00495	0.035664	0.407412	0.0423	0.0301

	family, response regulator AgrA	ko02024					
K07706	two-component system, LytTR family, sensor histidine kinase AgrC	ko02020, ko02024	M00495	0.03602	0.407602	0.0581	0.0384
K07487	transposase			0.037107	0.412455	0.0641	0.0523
K07124	uncharacterized protein			0.037476	0.413595	0.0584	0.054
K00549	5-methyltetrahydropteroyltriglutamate--homocysteine methyltransferase	ko00270, ko00450, ko01100, ko01110, ko01230	M00017	0.040538	0.430093	0.0936	0.086
K03496	chromosome partitioning protein			0.044662	0.444841	0.0807	0.0741
K21147	sulfur-carrier protein adenylyltransferase/sulfurtransferase	ko04122		0.045528	0.446162	0.0422	0.0477

Supplementary Table 4. Correlation between clinical symptoms and gut microbial abundance

**Feature**	**Bacteria**	**R square**	**P value**	**Q value**
UPDRS	Lactobacillus	0.21448	0.00002	0.0006
H&Y stage	Lactobacillus	0.2122	0.00002	0.0006
COMT inhibitor	Lactobacillus	0.1821	0.00002	0.0016
COMT inhibitor	Bifidobacterium	0.1721	0.00002	0.0016
Age	Prevotella	0.1521	0.00003	0.0016
UPDRS	Bifidobacterium	0.1352	0.00003	0.0016
Bristol scale	Prevotella	0.1318	0.00005	0.003
H&Y stage	Bifidobacterium	0.1292	0.00005	0.003
Constipation	Prevotella	0.12456	0.00007	0.003
Age	Prevotella	0.1193	0.00012	0.0057
Dysphagia	Bifidobacterium	0.1012	0.0003	0.012
Dysphagia	PAC001043	0.08509	0.00068	0.0155
Disease duration	Bifidobacterium	0.08401	0.00071	0.0163
Age	Parabacteroides	0.07936	0.0009	0.018
UPDR	PAC001043	0.07054	0.0014	0.0258
Age	Alistipes	0.06702	0.00167	0.0286
Amantadine	Lactobacillus	0.06343	0.00201	0.0301
Olfaction	Ruminococcus	0.0598	0.00241	0.0321
Disease duration	Lactobacillus	0.05773	0.00268	0.0338
H&Y stage	PAC001043	0.05613	0.0029	0.0348
Dysphagia	Roseburia	0.05288	0.00343	0.0376
Dysphagia	Lactobacillus	0.05264	0.00347	0.0378
COMT inhibitor	Oscillibacter	0.05187	0.00361	0.0392
H&Y stage	Sporobacter	0.04736	0.00454	0.0454
